# Quality of life: Seasonal fluctuation in Parkinson's disease

**DOI:** 10.3389/fneur.2022.1035721

**Published:** 2023-01-04

**Authors:** Lei Wu, Shiyu Li, Yilin Tang, Xiaoniu Liang, Zhiheng Xu, Tianyu Hu, Xiaoli Liu, Miao Cai, Xuedong Liu

**Affiliations:** ^1^Department of Neurology, The First Affiliated Hospital, Sun Yat-sen University; Guangdong Provincial Key Laboratory of Diagnosis and Treatment of Major Neurological Diseases; National Key Clinical Department and Key Discipline of Neurology, Guangzhou, China; ^2^State Key Laboratory of Medical Neurobiology, Department of Neurology, Huashan Hospital, National Research Center for Aging and Medicine and National Center for Neurological Disorders, Fudan University, Shanghai, China; ^3^Department of Neurology, Zhejiang Hospital, Hangzhou, China; ^4^Department of Neurology, Xijing Hospital, The Fourth Military Medical University, Xi'an, China

**Keywords:** Parkinson's disease, quality of life, seasons, epidemiologic factors, determinants

## Abstract

**Objective:**

Although the seasonal variation of motor and non-motor symptoms in Parkinson's disease (PD) has been reported, the association between seasonal change and quality of life in patients with Parkinson's disease remains to be explored.

**Methods:**

We recruited 1,036 patients with PD in this cross-sectional retrospective study. The patients were divided into four groups based on their date of assessment, according to the classical four seasons: group 1: March to May (*n* = 241); group 2: June to August (*n* = 259); group 3: September to November(*n* = 273); group 4: December to February (*n* = 263). The 39-item Parkinson's Disease Questionnaire (PDQ-39) and other clinical evaluation scales for motor and non-motor symptoms were administered. The determinants of the quality of life (QoL) were analyzed by multiple stepwise regression analyses.

**Results:**

A significant difference in PDQ-39 was found between group 1 (spring months) and group 3 (autumn months) after correction (*p* = 0.002). The Unified Parkinson's Disease Rating Scale part III (UPDRS-III) score was higher in group 1 (spring months) than in group 3 (the autumn months) (*p* = 0.033). The most severe determinant of QoL was the UPDRS-III score in group 1 and the Geriatric Depression Scale (GDS) score in groups 2, 3, and 4.

**Interpretation:**

The current study reported seasonal fluctuation of QoL in patients with PD, with higher scores during the spring months and lower scores in the autumn months. Since the determinants for QoL also vary by season, clinicians might need to focus on specific factors across seasons before initiating therapy.

## 1. Introduction

Parkinson's disease (PD) is a common neurodegenerative disorder characterized by bradykinesia, tremor, and rigidity (1). Both motor and non-motor symptoms occur in patients with PD. For motor symptoms, previous studies have shown a diurnal difference in severity, including changes in daily activity and a decrease in morning activities (2, 3).

Several previous studies have also worked on seasonal fluctuations in PD symptoms. Data from 546 patients with PD showed no difference in UPDRS motor scores over the traditional four seasons (4). Another study with 51 patients with PD reported no difference in hallucination severity with changing season (5). Van Wamelen et al. first explored the seasonal effect on non-motor symptoms (NMS) in 372 patients with PD in the United Kingdom and found worsening of NMS in the winter month compared with the summer month (6). Clear evidence for seasonal changes in the severity of PD symptoms is still lacking.

Quality of life (QoL) is a crucial outcome indicator in PD, and the improvement or maintenance of QoL is an important objective of treatment and care in patients with PD (7, 8). Both motor and non-motor symptoms make significant contributions to QoL (9, 10). However, hardly any QoL studies have compared QoL across different seasons in patients with PD. In addition, to take more precise pharmacological and non-pharmacological interventions to improve QoL, clinicians ought to explore the impact of clinical factors on QoL, according to seasonal variations in more detail. Therefore, we set out to determine the seasonal effect on QoL, and the potential different determinants of QoL across seasons in patients with PD in this study.

## 2. Methods

### 2.1 Subjects

Patients were recruited from the Department of Neurology of Huashan Hospital affiliated with Fudan University, Shanghai, China, from December 2011 to February 2021. Inclusion criteria were as follows: (1) a clinical diagnosis of idiopathic Parkinson's disease (iPD) according to the criteria of the United Kingdom PD Society Brain Bank (11) (2) age 45 years or older, and (3) consent to participate in the study. Cases with any history of stroke, disturbance of consciousness, epilepsy, traumatic brain injury, or severe psychiatric illness were excluded from the study. Cases missing clinical data (>20%) were excluded from the study.

The date when the patients performed the clinical assessments was recorded. Patients were assigned to one of the four groups, which were defined based on the classical four seasons. The classical seasons were defined as follows: group 1 (spring months): March to May; group 2 (summer months): June to August; group 3 (autumn months): September to November; and group 4 (winter months): December to February. Data on mean temperature were collected from the National Meteorological Information Center.

### 2.2 Standard protocol approvals, registrations, and patient consents

The study was approved by the Human Studies Institutional Review Board, Huashan Hospital, Fudan University. All patients provided their written informed consent in conformity with the Declaration of Helsinki to participate in our study.

### 2.3 Clinical assessments

Two physicians specializing in movement disorders performed clinical and neuropsychological tests. The Hoehn and Yahr stage (H&Y) (12) and the Unified Parkinson's Disease Rating Scale part III (UPDRS-III) were used to assess motor function during the wearing off of levodopa effect (off phase: at least 12 h off-medications) (13). The Mini-Mental State Examination (MMSE) (14) was used to evaluate global cognitive function. The Geriatric Depression Scale (GDS) (15) was applied to participants to assess depression and the Epworth Sleepiness Scale (ESS) (16) was used to evaluate one of the sleep disturbances, excessive daytime sleepiness. The Non-Motor Symptom Scale (NMSS) was used for the severity of non-motor symptoms (17, 18). The dosage of anti-parkinsonian drugs was converted into a total daily levodopa equivalent dose (LEDD) for standardization of the medications data (19).

QoL was assessed using the Parkinson's Disease Quality of Life Questionnaire (PDQ-39), including eight subdomains: mobility (10 items), activity of daily living (6 items), emotional well-being (6 items), stigma (4 items), social support (3 items), cognition (4 items), communication (3 items), and bodily discomfort (3 items) (20, 21). It is the most commonly used specific questionnaire for assessing QoL in patients with PD. Each item of the PDQ-39 is scored on a five-point Likert scale. In the current study, the PDQ-39 summary index (PDQ-39 SI) was standardized from the PDQ-39 original scores by dividing the scored points by the maximum possible points and then multiplying them by 100. The PDQ-39 SI ranges from 0 to 100, with higher scores representing worse QoL. All the questionnaires used in the study were validated in Chinese version. Any discrepant data were reviewed again by the investigators.

### 2.4 Statistical analysis

Categorical variables were expressed as frequencies (%), and continuous variables were expressed as the mean ± standard deviation (SD) or median (25%, 75%). Among the four groups (spring, summer, autumn, and winter), the chi-squared test was used for comparing the categorical variables, and the Kruskal–Wallis test or the one-way ANOVA test was used for comparing the continuous variables. The Mann–Whitney U-test was used for comparing the continuous variables between the two groups. For multiple comparison correction, the Bonferroni correction was used for the chi-squared test, and the Dwass, Steel, Critchlow-Fligner multiple comparison procedure (DSCF) was used for the Kruskal–Wallis test. Linear regression was used for correction to eliminate the effect of covariates factors. The correlations between the clinical characteristics and PDQ39 SI were analyzed by Spearman's rank correlation analysis. Multicollinearity test was used to choose relatively independent factors (VIF < 10) and estimate their standardized β regressive coefficient factors to uncover the main determinants of QoL in patients in different seasons with age, LEDD, GDS score, ESS score, MMSE score, NMSS score, and UPDRS-III scores entered. The R-squared (R^2^) index was used to determine the proportion of variance explained by the variables. Two-tailed *p*-values are presented. Differences were considered statistically significant at *P* < 0.05. The data analysis was conducted by SPSS version 26 (IBM SPSS Statistics for Windows, version 26.0. Armonk, NY: IBM Corp.).

## 3. Results

### 3.1 Demographic and clinical characteristics

A total of 1,207 patients who were diagnosed with iPD were recruited. After that, 171 subjects were excluded according to the specified study exclusion criteria (Supplementary Figure 1), and the remaining 1,036 patients were included: 241 patients allocated to group 1 (March to May), 259 to group 2 (June to August), 273 to group 3 (September to November), and 263 to group 4 (December to February). The clinical characteristics of the patients are shown in Table 1. Data for the mean temperature of each month were collected from the National Meteorological Center and were in accordance with the division of the seasonal groups (Supplementary Figure 2). There were no significant differences in age, gender, disease duration, and LEDD among the four groups. Regarding motor symptoms, no differences were found in the H&Y stage. For UPDRS-III scores, group 1 was significantly higher compared with group 3 (*p* = 0.005). No difference was observed in MMSE, GDS, ESS, and NMSS scores among the four groups (Table 1). There were no significant differences in NMSS domains among the four groups (Supplementary Table 1).

**Table 1:** Demographics and seasonal variations for PDQ-39 and other scale results.

	**Group 1 (*n =* 241)**	**Group 2 (*n =* 259)**	**Group 3 (*n =* 273)**	**Group 4 (*n =* 263)**	*P*-value^*^
**Demographics**					
Age	61.3 (8.03)	60.4 (8.17)	61.2 (7.78)	61.2 (8.70)	0.585^#^
Gender	130/111 (53.94%M)	149/110 (57.53%M)	170/103 (62.27%M)	141/122 (53.61%M)	0.153
duration	51.0 (21.0,118.0)	54.0 (20.0,103.0)	44.0 (20.0,97.0)	47.0 (23.5,85.5)	0.530
HY level	2.41 (1.08)	2.33 (0.94)	2.29 (0.98)	2.21 (0.94)	0.150
LEDD	550.0 (315.0,826.0)	500.0 (300.0,800.0)	450.0 (300.0,774.0)	450.0 (288.0,708.0)	0.058
PDQ39	42.1 (27.4)	39.5 (28.8)	34.6 (27.8)	37.3 (27.9)	0.049^*^ (season1-3 *p =* 0.040^†^)
Mobility	12.7 (11.0)	11.2 (10.4)	9.28 (9.62)	10.1 (10.2)	0.033^*^ (season1-3 *p =* 0.010^†^)
Daily activities	6.15 (5.91)	5.70 (5.75)	4.94 (5.65)	5.65 (6.09)	0.064
Emotional well-being	6.70 (5.31)	6.46 (5.83)	5.84 (5.51)	6.14 (5.56)	0.124
Stigma	4.46 (4.21)	4.27 (4.29)	3.87 (4.00)	4.08 (4.31)	0.357
Social support	1.28 (2.00)	1.32 (1.98)	1.13 (2.07)	1.15 (1.85)	0.233
Cognition	4.87 (3.26)	4.83 (3.56)	4.52 (3.35)	4.67 (3.56)	0.493
Communication	2.30 (2.63)	2.21 (2.60)	1.94 (2.72)	2.02 (2.58)	0.088
Bodily discomfort	3.80 (2.95)	3.49 (2.91)	3.11 (2.77)	3.46 (2.94)	0.050 (season1–3 *p =* 0.028^†^)
GDS	12.8 (7.41)	11.6 (7.64)	11.7 (7.71)	11.7 (7.07)	0.193
MMSE	25.8 (4.12)	26.1 (3.97)	26.5 (3.72)	26.2 (4.28)	0.208
ESS	6.78 (5.05)	6.78 (5.14)	6.21 (4.46)	6.06 (4.78)	0.350
NMSS	12.5 (6.75)	12.2 (6.94)	12.1 (6.62)	11.9 (6.82)	0.672
UPDRSIII_OFF	36.5 (16.6)	33.4 (16.6)	30.0 (17.5)	31.5 (14.7)	0.005^*^ (season1–3 *p =* 0.003)

Group 1: spring month (March to May); Group 2: summer month (June to August); Group 3: autumn month (September to November), and Group 4: winter month (December to February).

HY level, Hoehn–Yahr classification level; LEDD, levodopa equivalent dose; PDQ, Parkinson's Disease Questionnaire; GDS, Geriatric Depression Rating Scale; MMSE, Mini-Mental State Examination; ESS, Epworth Sleepiness Scale; NMSS, Non-Motor Symptom Scale; UPDRS, Unified Parkinson's Disease Rating Scale.

The data of disease duration and levodopa equivalent dose are presented as median (25%, 75%) or as mean ± SD.

* Comparison among the four groups with Group 1, Group 2, Group 3, and Group 4.

# The categorical variables were compared among the four groups using the chi-squared test.

† The Dwass, Steel, Critchlow-Fligner multiple comparison procedure (DSCF) was used for the Kruskal–Wallis test among the four groups.

The continuous variables were compared among the four groups using the Kruskal–Wallis test.

### 3.2 QoL assessment

The results of QoL assessed by the PDQ-39 are shown in Table 1. Group 1 had a significantly higher PDQ-39 score in comparison with group 3 (*p* = 0.002). After adjustment for GDS and UPDRS-III scores, PDQ-39 scores were still significantly higher in group 1 compared with group 3 (*p* = 0.040). For the PDQ-39 subdomains, we observed a significant distinct difference in the mobility subdomain (*p* = 0.010) and bodily discomfort subdomain (*p* = 0.028) between group 1 and group 3 (Table 1) after adjustment. No difference was observed in the remaining PDQ-39 subdomains.

### 3.3 Determinants of QoL

Correlation analysis between clinical characteristics and PDQ39 SI was performed in all patients with PD (Supplementary Table 2). To reveal the determinants of QoL in patients with PD, we conducted a multiple stepwise analysis in four seasonal groups with age, LEDD, UPDRS-III scores, GDS scores, MMSE scores, ESS scores, and NMSS scores entered (Table 2). The most severe determinant of the PDQ-39 was UPDRS-III scores in group 1 (R^2^ = 0.3240, β = 0.3480, *P* < 0.001), and GDS scores in group 2 (R^2^ = 0.4970, β = 0.3380, *P* < 0.001), group 3 (R^2^ = 0.4640, β = 0.3640, *P* < 0.001), and group 4 (R^2^ = 0.4610, β = 0.3970, *P* < 0.001). For NMSS domains, perceptual disturbances and hallucinations was the most severe determinants in group 1 (R^2^ = 0.2540, β = 0.2650, *P* < 0.001) and group 3 (R^2^ = 0.2320, β = 0.2120, *P* < 0.001), and gastrointestinal symptoms is the most severe determinant in group 2 (R^2^ = 0.2460, β = 0.1690, *P* = 0.012) and group 4 (R^2^ = 0.2550, β = 0.2320, *P* < 0.001) (Supplementary Table 3).

**Table 2:** Multiple stepwise analysis of determinants in four seasonal groups.

	**R^2^**	**β**	**95% Confidence interval**	**p**
**Total**				
Duration	0.1220	0.1220	(0.025, 0.081)	< 0.001
MMSE	0.0740	−0.0739	(−0.915, −0.128)	0.010
ESS	0.1610	0.0982	(0.236, 0.877)	< 0.001
NMSS	0.3750	0.1962	(0.581, 1.183)	< 0.001
GDS	0.4490	0.3695	(1.139, 1.610)	< 0.001
UPDRSIII_OFF	0.3580	0.2867	(0.378, 0.588)	< 0.001
**Group 1**				
NMSS	0.3550	0.2795	(0.564, 1.858)	< 0.001
GDS	0.3620	0.3239	(0.625, 1.56)	< 0.001
UPDRSIII_OFF	0.3260	0.3783	(0.411,0. 819)	< 0.001
**Group 2**				
ESS	0.2400	0.1629	(0.262, 1.510)	0.006
NMSS	0.4280	0.1909	(0.228, 1.376)	0.007
UPDRSIII_OFF	0.4170	0.3131	(0.325, 0.734)	< 0.001
GDS	0.4990	0.3477	(0.801, 1.756)	< 0.001
**Group 3**				
UPDRSIII_OFF	0.3250	0.2018	(0.112, 0.574)	0.004
NMSS	0.3590	0.2176	(0.402, 1.514)	0.002
GDS	0.4660	0.3709	(0.938, 2.001)	< 0.001
**Group 4**				
NMSS	0.3640	0.1414	(0.044, 1.174)	0.035
UPDRSIII_OFF	0.3410	0.2478	(0.193, 0.658)	< 0.001
GDS	0.4630	0.4156	(1.089, 2.031)	< 0.001

MMSE, Mini–Mental State Examination; ESS, Epworth Sleepiness Scale; NMSS, Non–Motor Symptom Scale; GDS, Geriatric Depression Rating Scale; UPDRS, Unified Parkinson's Disease Rating Scale.

Linear regression was used for correction to eliminate the effect of covariate factors.

The proportion of variance was presented as the R–squared (R^2^) index. The coefficient factors were presented as β (standardized coefficients β).

## 4. Discussion

To the best of our knowledge, this is the first study to explore the seasonal variation of QoL in patients with PD. We observed that QoL was more impaired in the spring months compared with the autumn months after adjusting for UPDRS-III scores. Furthermore, motor function was considered to be the most crucial determinant for QoL in the spring months, while depression was indicated to be the most vital determinant for the summer, autumn, and winter months (22).

Why was QoL worse in the spring months? Seasonal variations in QoL might be due to the motor and non-motor symptoms (23). These symptoms can be influenced by many factors, including environmental aspects and lifestyle changes (21).

For example, humidity may play a role in seasonal variations. Previous studies found that high indoor humidity makes poor air quality, leading to a higher risk of reduced dopamine levels and dopamine neuron degeneration at home (24, 25). The higher humidity level in the spring months might thus aggravate the severity of motor symptoms, finally deteriorating the PDQ-39 scores (26). Circadian rhythm also influenced motor and non-motor symptoms in patients with PD by suprachiasmatic nuclei (SCN), as was suggested by several studies (27, 28). Seasonal allergies were associated with PD, as the immune system was found to be related to PD (29). Previous studies indicated that PD was positively associated with allergic rhinitis (30, 31), especially with allergies to plants and antibiotics (32). Also, seasonal influenza was reported to be associated with the severity of PD symptoms, which might appear more frequently in the spring months, thus influencing the results (33). Therefore, these environmental changes might influence patients with PD, reflecting on QoL outcomes.

In addition, spring break activities (visiting parents, vacationing with friends, heavy drinking, etc.) dramatically increased during and after the long Spring Festival holiday in China. For patients with PD, the inability to participate in physical activity might lead to psychological discrepancy and aggravate neuropsychiatric symptoms, such as anxiety and depression, thereby worsening QoL. The reason why QoL was worse in the spring months remains to be explored; however, we suggest that worsening Qol in patients with PD might result from a mix of several factors, rather than a single one (34).

Previous studies have focused on the seasonal fluctuation of motor and non-motor symptoms in patients with PD. The seasonal effect was found on non-motor symptoms in 372 patients with PD and worsening conditions in the winter months compared with the summer months (6). Our study, however, did not observe similar seasonal variations in NMS. It is speculated that our patients showed less severe non-motor symptoms, so the fluctuation of symptom severity was not remarkable. A study for 1,005 patients was conducted in China (35) to explore the annual cycle with the severity of NMS for patients with PD in Southeast China (35) and found differences mainly in sleep parameters such as sleep efficiency and sleep stages recorded by polysomnography (PSG). The study also showed no difference in MMSE, NMSQ, ESS, and depression scores, which was consistent with our results.

Diabetes mellitus, as a concomitant disease, was suggested to be a determinant of PD risk and progression according to previous studies (36). Nevertheless, no significant difference was shown in PDQ-39 scores between our patients with underlying diseases, such as diabetes and high blood pressure (HBP), and there was no significant difference in contribution among the four groups (Supplementary Table 4). Therefore, it was suggested that concomitant disease in our study groups should be unlikely to explain the seasonal Qol difference. We further explore the impact of the possible influencing factors of QoL across seasons. A series of previous studies demonstrated that depression was the main and most frequently identified contributor to QoL in both patients with PD and health controls (37). In line with previous studies, we found that depression was the most important determinant for QoL in the summer, autumn, and winter months. Interestingly, when it comes to the spring months, the UPDRS-III scores, which represent the severity of motor symptoms, accounted for the most critical determinant of QoL in patients with PD. As discussed earlier, the increased need for physical activity during the spring months might partly contribute to this result that motor symptoms impose a greater impact on QoL than other factors. Our results suggest that the determinants for QoL vary by season. Therefore, to maximize the benefits of QoL, clinicians might focus on specific factors based on seasonal variations when initiating pharmacological or non-pharmacological interventions.

The study contained a relatively large capacity of enrolled patients with PD and a full set of clinical and neuropsychological assessments. However, there are still certain limitations in our study. The main limitation of the research is the cross-sectional design, which could not analyze the longitudinal impacts of these factors on QoL and make causal inferences. Furthermore, a prefer study was considered to take place with one patient throughout the year. However, if we follow up on QoL at different times within individual patients with PD, we cannot exclude the possibility that the ameliorating or worsening of QoL resulted primarily from the progression of the underlying disease. Moreover, the enrolled patients in the current study showed no difference in demographic information, which indicates that seasonal changes might play the most important role in QoL fluctuation.

## 5. Conclusion

Taken together, the current study reported seasonal differences in QoL in patients with PD, with higher scores during the spring months and lower scores during the autumn months. Depression imposed the greatest impact on QoL during the summer, autumn, and winter seasons, while motor symptoms turned out to be the crucial factor in the spring months. The findings need to be confirmed by further research with cohort studies and larger sample populations.

## Data availability statement

The raw data supporting the conclusions of this article will be made available by the authors, without undue reservation.

## Ethics statement

Ethical review and approval was not required for the study on human participants in accordance with the local legislation and institutional requirements. Written informed consent from the patients/participants legal guardian/next of kin was not required to participate in this study in accordance with the national legislation and the institutional requirements.

## Author contributions

MC and XuL contributed to designing and conceptualization of the study. LW, SL, YT, XiaolL, XiaonL, ZX, and TH were involved in data collection. SL, YT, and XiaonL participated in the statistical analysis of data. LW, SL, MC, and XuL were involved in drafting and revising the manuscript for intellectual content. All authors contributed to the article and approved the submitted version.
