# Supplementary material for: Quality of life: Seasonal fluctuation in Parkinson's disease
Source: Front Neurol. 2023 Jan 4;13:1035721. doi: 10.3389/fneur.2022.1035721 (PMC9846796; doi:10.3389/fneur.2022.1035721)
Supplement: Supplementary file 1 [file Table_1.docx]

***Supplementary Table 1 Seasonal variations for NMSS domains.***

|  | **Group1(n=241)** | **Group2(n=259)** | **Group3(n=273)** | **Group4(n=263)** | **P value*** |
| --- | --- | --- | --- | --- | --- |
| **NMSS** | 12.5(6.75) | 12.2(6.94) | 12.1(6.62) | 11.9(6.82) | 0.672 |
| Cardiovascular/falls | 0.717(0.701) | 0.694(0.663) | 0.751(0.722) | 0.694 (0.676) | 0.844 |
| Sleep/fatigue | 1.52 (1.21) | 1.68(1.34) | 1.60(1.33) | 1.44 (1.31) | 0.167 |
| Mood/cognition | 2.95 (1.75) | 2.90 (1.85) | 2.80(1.84) | 2.77 (1.74) | 0.690 |
| Perceptual/hallucinations | 0.906 (1.00) | 0.948 (1.08) | 0.806 (0.995) | 0.933 (1.03) | 0.343 |
| Attention/memory | 1.45 (1.10) | 1.41 (1.14) | 1.38 (1.14) | 1.44 (1.11) | 0.869 |
| Gastrointestinal | 0.934 (0.983) | 1.08(0.993) | 0.958 (0.924) | 1.09 (0.960) | 0.122 |
| Urinary | 1.37(1.09) | 1.35 (1.12) | 1.47(1.15) | 1.37 (1.11) | 0.591 |
| Sexual | 0.971 (0.833) | 0.867 (0.850) | 0.903 (0.823) | 0.867 (0.868) | 0.433 |
| Miscellaneous | 1.45 (1.22) | 1.40 (1.21) | 1.45 (1.16) | 1.29 (1.20) | 0.295 |

NMSS, Non-Motor Symptom Scale. The data are presented as mean ± SD.

Note: *Comparison among the four groups with Group1, Group2, Group3 and Group4. The continuous variables were compared among the four groups by Kruskal-Wallis test.
