# Supplementary material for: Quality of life: Seasonal fluctuation in Parkinson's disease
Source: Front Neurol. 2023 Jan 4;13:1035721. doi: 10.3389/fneur.2022.1035721 (PMC9846796; doi:10.3389/fneur.2022.1035721)
Supplement: Supplementary file 2 [file Table_2.docx]

***Supplementary Table2.* Correlation analysis between clinical characteristics and PDQ39 SI.**

|  |  | Age | Duration | LEDD | MMSE | ESS | GDS | NMSS | UPDRS Ⅲ__OFF | PDQ39 |
| --- | --- | --- | --- | --- | --- | --- | --- | --- | --- | --- |
| Age | Spearman’s rho | — |  |  |  |  |  |  |  |  |
|  | p-value | — |  |  |  |  |  |  |  |  |
| Duration | Spearman’s rho | 0.098 ** | — |  |  |  |  |  |  |  |
|  | p-value | 0.002 | — |  |  |  |  |  |  |  |
| LEDD | Spearman’s rho | 0.033 | 0.580*** | — |  |  |  |  |  |  |
|  | p-value | 0.341 | < .001 | — |  |  |  |  |  |  |
| MMSE | Spearman’s rho | -0.177*** | -0.135*** | -0.192*** | — |  |  |  |  |  |
|  | p-value | < .001 | < .001 | < .001 | — |  |  |  |  |  |
| ESS | Spearman’s rho | -0.016 | 0.253*** | 0.286*** | -0.101** | — |  |  |  |  |
|  | p-value | 0.603 | < .001 | < .001 | 0.001 | — |  |  |  |  |
| GDS | Spearman’s rho | -0.007 | 0.248*** | 0.221*** | -0.197*** | 0.311*** | — |  |  |  |
|  | p-value | 0.835 | < .001 | < .001 | < .001 | < .001 | — |  |  |  |
| NMSS | Spearman’s rho | 0.069* | 0.339*** | 0.380*** | -0.200*** | 0.428*** | 0.593*** | — |  |  |
|  | p-value | 0.027 | < .001 | < .001 | < .001 | < .001 | < .001 | — |  |  |
| UPDRS Ⅲ__OFF | Spearman’s rho | 0.067 | 0.577*** | 0.446*** | -0.317*** | 0.235*** | 0.336*** | 0.391*** | — |  |
|  | p-value | 0.078 | < .001 | < .001 | < .001 | < .001 | < .001 | < .001 | — |  |
| PDQ39 | Spearman’s rho | 0.086** | 0.493*** | 0.422*** | -0.252*** | 0.396*** | 0.681*** | 0.653*** | 0.614*** | — |
|  | p-value | 0.005 | < .001 | < .001 | < .001 | < .001 | < .001 | < .001 | < .001 | — |

Note: Spearman's rank correlation coefficient was used to test the correlation between the clinical characteristics and PDQ39 SI.

**: P＜0.01

***: P＜0.001
