# Supplementary material for: Quality of life: Seasonal fluctuation in Parkinson's disease
Source: Front Neurol. 2023 Jan 4;13:1035721. doi: 10.3389/fneur.2022.1035721 (PMC9846796; doi:10.3389/fneur.2022.1035721)
Supplement: Supplementary file 3 [file Table_3.docx]

***Supplementary Table 3 Multiple stepwise analysis of determinants in four seasonal groups for NMSS domains.***

|  | **R**2 | **β** | p |
| --- | --- | --- | --- |
| **Group1** |  |  |  |
| Perceptual/hallucinations | 0.2540 | 0.2650 | <0.001 |
| Urinary | 0.1600 | 0.1280 | 0.046 |
| **Group2** |  |  |  |
| Sleep/fatigue | 0.2080 | 0.1410 | 0.041 |
| Gastrointestinal | 0.2460 | 0.1690 | 0.012 |
| **Group3** |  |  |  |
| Perceptual/hallucinations | 0.2320 | 0.2120 | <0.001 |
| Gastrointestinal | 0.1970 | 0.1590 | 0.006 |
| Sexual | 0.1660 | 0.1400 | 0.015 |
| **Group4** |  |  |  |
| Sleep/fatigue | 0.1690 | 0.1390 | 0.026 |
| Attention | 0.1870 | 0.1440 | 0.019 |
| Gastrointestinal | 0.2550 | 0.2320 | <0.001 |
| Miscellaneous | 0.2450 | 0.2110 | 0.002 |

NMSS, Non-Motor Symptom Scale

Note: Linear regression was used for correction to eliminate the effect of covariates factors. The proportion of variance was presented as the R-squared (R^2^) index. The coefficient factors were presented as β (standardized coefficients β).
