# Supplementary material for: Quality of life: Seasonal fluctuation in Parkinson's disease
Source: Front Neurol. 2023 Jan 4;13:1035721. doi: 10.3389/fneur.2022.1035721 (PMC9846796; doi:10.3389/fneur.2022.1035721)
Supplement: Supplementary file 4 [file Table_4.docx]

***Supplementary Table 4 Distribution for underlying diseases in different seasonal groups and Qol outcomes.***

|  | **Number** | **PDQ39** | P* |
| --- | --- | --- | --- |
| **Group1 (n=241)** |  |  |  |
| Diabetes | 19 | 45.42(25.42) | 0.536 |
| Non-diabetes | 222 | 41.31(27.96) |  |
| HBP | 65 | 36.77(25.35) | 0.100 |
| nHBP | 176 | 43.43(28.43) |  |
| **Group2 (n=258)** |  |  |  |
| Diabetes | 20 | 39.97(29.11) | 0.789 |
| Non-diabetes | 238 | 38.15(31.52) |  |
| HBP | 59 | 40.58(27.91) | 0.825 |
| nHBP | 199 | 39.61(29.69) |  |
| **Group3 (n=273)** |  |  |  |
| Diabetes | 25 | 35.76(29.05) | 0.753 |
| Non-diabetes | 248 | 33.94(27.27) |  |
| HBP | 69 | 34.54(24.33) | 0.881 |
| nHBP | 204 | 33.97(28.40) |  |
| **Group4 (n=262)** |  |  |  |
| Diabetes | 22 | 41.73(29.90) | 0.643 |
| Non-diabetes | 240 | 38.74(28.80) |  |
| HBP | 64 | 36.34(29.37) | 0.399 |
| nHBP | 198 | 39.85(28.70) |  |

Group1: spring month (March to May); Group2: summer month (June to August); Group3: autumn month (September to November); Group4: winter month (December to February)

Qol, quality of life; HBP, high blood pressure. The data are presented as mean ± SD.

Note: *The continuous variables were compared between the two disease groups (diabetes to non-diabetes, HBP to nHBP) in different seasons by Mann-Whitney U test.
