# Supplementary figures and images for: Quality of life: Seasonal fluctuation in Parkinson's disease
Source: Front Neurol. 2023 Jan 4;13:1035721. doi: 10.3389/fneur.2022.1035721 (PMC9846796; doi:10.3389/fneur.2022.1035721)

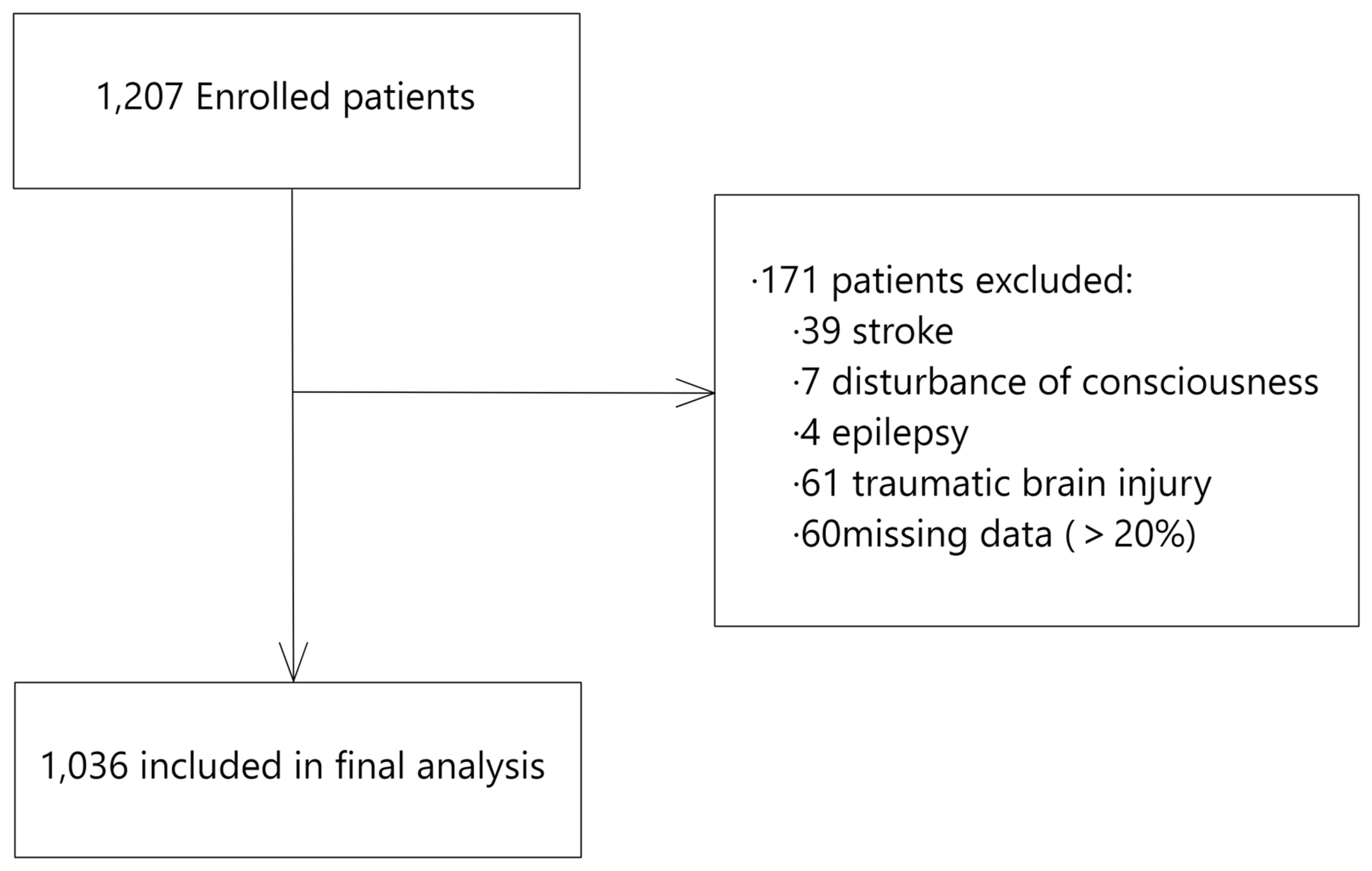

Supplement: Supplementary file 5 [file Image_1.png]

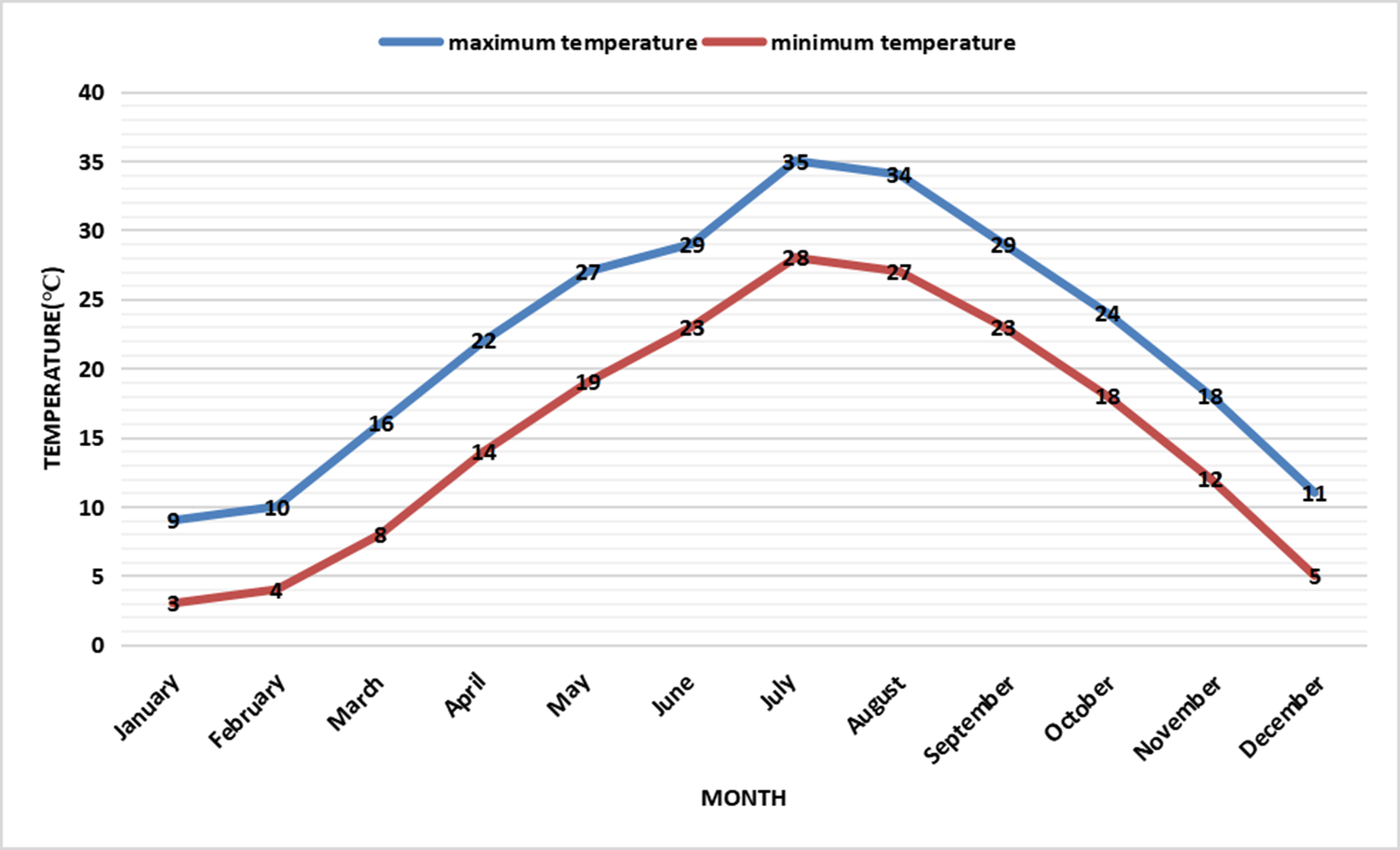

Supplement: Supplementary file 6 [file Image_2.png]
